# Characterising sex differences of autosomal DNA methylation in whole blood using the Illumina EPIC array

**DOI:** 10.1186/s13148-022-01279-7

**Published:** 2022-05-14

**Authors:** Olivia A. Grant, Yucheng Wang, Meena Kumari, Nicolae Radu Zabet, Leonard Schalkwyk

**Affiliations:** 1 School of Life Sciences, University of Essex, Colchester, CO4 3SQ UK; 2 Institute of Social and Economic Research, University of Essex, Colchester, CO4 3SQ UK; 3 Blizard Institute, Barts and The London School of Medicine and Dentistry, Queen Mary University of London, London, E1 2AT UK; 4 School of Computer Science and Electronic Engineering, University of Essex, Colchester, CO4 3SQ UK

**Keywords:** Epigenetics, DNA methylation, Gene regulation, Autosomes, Sex differences, Sex, Illumina EPIC array

## Abstract

**Background:**

Sex differences are known to play a role in disease aetiology, progression and outcome. Previous studies have revealed autosomal epigenetic differences between males and females in some tissues, including differences in DNA methylation patterns. Here, we report for the first time an analysis of autosomal sex differences in DNAme using the Illumina EPIC array in human whole blood by performing a discovery (*n* = 1171) and validation (*n* = 2471) analysis.

**Results:**

We identified and validated 396 sex-associated differentially methylated CpG sites (saDMPs) with the majority found to be female-biased CpGs (74%). These saDMP’s are enriched in CpG islands and CpG shores and located preferentially at 5’UTRs, 3’UTRs and enhancers. Additionally, we identified 266 significant sex-associated differentially methylated regions overlapping genes, which have previously been shown to exhibit epigenetic sex differences, and novel genes. Transcription factor binding site enrichment revealed enrichment of transcription factors related to critical developmental processes and sex determination such as SRY and ESR1.

**Conclusion:**

Our study reports a reliable catalogue of sex-associated CpG sites and elucidates several characteristics of these sites using large-scale discovery and validation data sets. This resource will benefit future studies aiming to investigate sex specific epigenetic signatures and further our understanding of the role of DNA methylation in sex differences in human whole blood.

**Supplementary Information:**

The online version contains supplementary material available at 10.1186/s13148-022-01279-7.

## Introduction

Sex is an important covariate in all epigenetic research due to its role in the incidence, progression and outcome of many phenotypic characteristics and human diseases [1, 2]. There is an increasing interest as to which role epigenetic modifications (such as DNA methylation) may play in the underpinnings for relationships between environmental exposures and disease onset. In addition, sex has previously been shown to have a strong influence on DNA methylation variation [3–7]. However, the idea that DNA methylation variation between males and females may underlie the sex biases observed in diseases has not been well documented thus far.

Sex differences in disease prevalence are sometimes explained at the molecular level and rooted in genetic differences between males and females. Differences in sex chromosome complement have independently been shown to direct differences in gene expression and chromatin organisation [8–11]. Furthermore, these differences in sex chromosome complement are sufficient to explain sex bias seen in some diseases. For example, X chromosome number has previously been shown to impact immune cell population and occasionally therefore the development of diseases such as autoimmunity [12, 13].

Previous research has also revealed sex differences in gene expression of autosomal genes as well as sex chromosome linked genes [14]. It is worth noting that most of the differences in gene expression on the autosomes are small differences [15]. However, small expression differences may still be associated with great effects on phenotypic characteristics and disease incidence and onset. Others also identified sex differences in chromatin accessibility and histone modifications, thus suggesting that different epigenetic factors contribute to gene expression sex biases seen in some diseases [16].

Sex specific gene expression and levels of sex hormones may be mediated by epigenetic mechanisms, including DNA methylation. Several genome wide association methylome studies (or Epigenome Wide Association Studies, EWAS) have highlighted differences in DNA methylation patterns linked to sex differences in genes on the autosomes [15–18]. Previous studies have reported sites and regions showing varying methylation due to sex differences in several tissues such as saliva, placenta, brain, pancreatic islets and whole blood [15, 17, 19–28]. These studies highlight the presence of autosomal loci displaying sex-biased DNA methylation patterns across the genome for several tissues. In order to determine their role in disease and developmental processes, these loci warrant further exploration.

However, due to X chromosome inactivation in females, large differences in methylation levels of X chromosomes can be observed between males and females [29]. Recent research suggests that normalising methylation data with the sex chromosomes introduces a large technical bias to many autosomal CpGs [30]. This technical bias has been reported to result in many autosomal CpG sites being falsely associated with sex even when male and female samples are normalised independently of each other, a method employed by some studies in the field. Moreover, it also leads to many autosomal CpGs being incorrectly identified to be more methylated in male samples compared to female samples. Therefore, the breadth of autosomal DNA methylation variation between males and females is still not well understood and requires further clarification. Extra steps were therefore employed in this study by applying a normalisation method which aims to reduce bias introduced to autosomal CpGs [30] to uncover true biological differences and determine patterns of global DNA methylation levels between males and females.

Additionally, it is worth noting that thousands of autosomal CpGs do show very small differences in DNA methylation patterns between males and females. However, a robust and well-annotated catalogue of sites showing the largest differences still needs to be characterised.

Here, we use the EPIC BeadChip to assess autosomal sex differences in DNA methylation levels from whole blood at individual sites and genomic regions. All individuals involved in this study were part of Understanding Society: The UK Household longitudinal study [31]. Additionally, we adequately handle the technical bias introduced by sex chromosomes. To our knowledge, this is the largest study using the Illumina EPIC BeadChip (allowing for interrogation of ~ 850,000 sites across the genome) to investigate autosomal sex differences in DNA methylation at CpG sites in whole blood.

## Results

### Females show higher methylation at a subset of autosomal loci

Analysis of DNA methylation (DNAme) differences between males and females on the autosomes was performed using linear regression for the Illumina EPIC BeadChip for 1171 individuals (682 females and 489 males) for discovery and repeated in a validation data set of 2471 participants (1339 females and 1132 males). After data processing and cleaning, *n* = 747,302 CpGs were analysed (see *Material and Methods*). Sites which are known SNP probes, cross hybridising or X/Y linked probes were excluded. Moreover, since whole blood is a bulk tissue, we calculated the estimated cell type proportions for whole blood between our male and female samples to assess whether any differences in cell type proportions would potentially be reflected in our results resulting in false positives. Using Wilcoxon test, we found no significant difference in the proportions of Granulocytes between males and females, but we did find statistically significant differences in proportions of CD4T, CD8T, Natural killer, B cells and monocytes (Additional file 6: Figure S1B and S1D). We therefore included cell type proportions in our models for identifying sex-associated differentially methylated probes and regions. After adjusting for multiple testing using the Benjamini–Hochberg FDR method (FDR *p* < 0.05), we identified 54,261 autosomal CpGs associated with sex in our discovery and validation data set (Additional file 6: Figure S1C). Of those CpGs, 60% (33,103 CpGs) were more highly methylated in females and the remaining 40% (21,788 CpGs) were more methylated in males. Gene ontology analyses showed several enriched terms for these 54,261 autosomal CpGs (Table 1) which included terms related to mammalian sex determination and gonad development, specifically several signalling pathways such as Ras signalling, MAPK signalling, Wnt and Hippo signalling [32–1]. Other terms included pathways related to cancer and cellular proliferation (Table 35). This is not surprising though, as there is overwhelming evidence that sex influences cancer risk, progression, and treatment response [36–38]. It is also now well accepted that sex differences may significantly impact on the cell biology of cancer [39]. Further, epigenetic dysregulation is also now accepted widely as a mechanism for cancer initiation and progression. This may be through transcriptional activation or repression of specific autosomal loci through means of DNA methylation. Therefore, one can hypothesise that sex specific patterns may influence the ability of cancer cells to adopt a stem cell like phenotype. This enables us to draw a link between epigenetic signatures and cancer pathways. It is likely that these sex differences in DNA methylation in part cause or are caused by differing levels of sex hormones such as androgen or oestrogen. This idea is supported by previous literature highlighting that DNA methylation transcriptionally represses masculinising genes and that this depends on gonadal hormones during development [39].

**Table 1:** Enriched GO terms among the 54,261 CpGs identified to be significantly associated with sex

Path	Description	*N*	DE	P.DE	FDR
path:hsa04020	Calcium signalling pathway	240	192	4.10E−09	1.41E−06
path:hsa04015	Rap1 signalling pathway	210	169	1.15E−07	1.98E−05
path:hsa05200	Pathways in cancer	531	382.8333	1.91E−07	2.19E−05
path:hsa04014	Ras signalling pathway	232	180	2.97E−07	2.56E−05
path:hsa04010	MAPK signalling pathway	294	223.33333	2.24E−06	0.00015382
path:hsa04360	Axon guidance	182	148.5	3.46E−06	0.00019826
path:hsa04072	Phospholipase D signalling pathway	148	121	4.51E−06	0.00022149
path:hsa04310	Wnt signalling pathway	166	129.5	7.50E−05	0.00322397
path:hsa04371	Apelin signalling pathway	139	107.5	0.00011119	0.00363348
path:hsa04724	Glutamatergic synapse	114	93	0.00011675	0.00363348
path:hsa04390	Hippo signalling pathway	157	123.5	0.00012001	0.00363348
path:hsa01521	EGFR tyrosine kinase inhibitor resistance	79	67.5	0.00013811	0.00363348
path:hsa04071	Sphingolipid signalling pathway	119	94.5	0.00013944	0.00363348
path:hsa05226	Gastric cancer	149	115.5	0.00014787	0.00363348
path:hsa04550	Signalling pathways regulating pluripotency of stem cells	143	108.5	0.00059275	0.01359379
path:hsa04151	PI3K-Akt signalling pathway	354	245	0.00076003	0.01634056
path:hsa05224	Breast cancer	147	111.5	0.00092102	0.01863701
path:hsa04725	Cholinergic synapse	113	88	0.00148428	0.02836619
path:hsa04961	Endocrine and other factor-regulated calcium reabsorption	53	44	0.00209794	0.03798375
path:hsa05225	Hepatocellular carcinoma	168	123.5	0.00284431	0.04892215

*N* indicates the number of genes in the KEGG term. DE refers to the number of genes annotated to the sex-associated DMPs which are differentially methylated. P.DE indicates the *P* value for over representation of the KEGG term in this data set. FDR indicates the false discovery rate (using the Benjamini and Hochberg method)

**Fig. 1:**
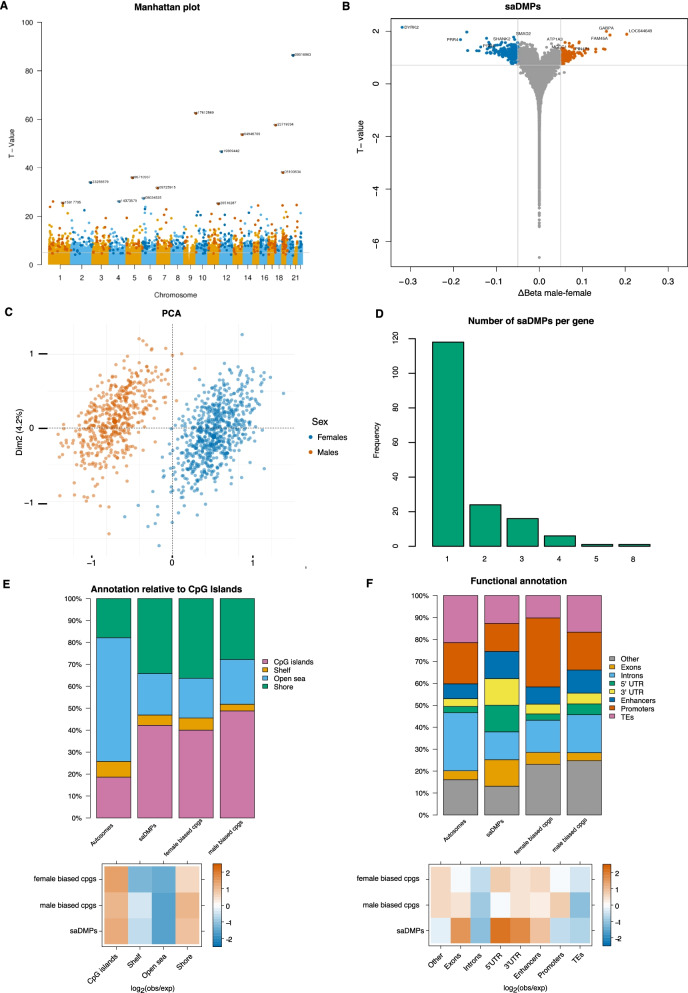
Location and characterisation of saDMPs. **A** Manhattan plot for EWAS analysis of sex. CpG sites which met a threshold of FDR < 0.05 and had an average beta change of > 0.05 and found in both discovery and validation data sets were considered significant and are represented by darker colours. **B** Volcano plot for saDMPs. CpGs which are not significant in both the discovery and validation data sets are represented in grey, replicated saDMPs male-biased CpGs are in orange and replicated saDMPs female-biased CpGs in blue. Grey points displayed beyond the cut off points represent CpG sites which were met the criteria in the discovery data set (FDR < 0.05 and deltaBeta value > than 0.05 in any direction) but were not replicated in the validation data set. **C** Principal component analysis of beta values at the significant saDMPs. Male samples are indicated in orange while female samples are indicated in blue. **D** Number of saDMPs harboured by individual genes. **E** Top panel shows the annotation of all saDMPs (*n* = 396), female-biased CpGs (*n* = 293) and male-biased CpGs (*n* = 103) relative to CpG island regions compared to the autosomal background. Bottom panel shows the log_2_ (obs/exp) annotations based on the autosomal background of the different annotations. **F** Top panel shows the overlap of all saDMPs (*n* = 396), female-biased CpGs (*n* = 293) and male-biased CpGs (*n* = 103) with genomic features compared to the autosomal background. Bottom panel shows the log_2_ (obs/exp) annotations based on the autosomal background of the different annotations

The lambda value of the Q-Q plots is slightly high (Additional file 6: Figure S1A and S1C) indicating slight inflation of test statistics and, in order to ensure we detect true sex differences, we selected CpGs that displayed large differences in methylation. Further, as a high proportion of CpG sites across the genome, we were also interested in investigating further, those CpG sites which show the largest differences between sexes. Thus, we further filtered our list of 54,261 CpGs by only considering those probes that displayed the largest sex differences, determined by a ΔBeta value (absolute difference between average Beta values in male and female samples) greater than 0.05. A total of 396 CpGs met this criterion (called sex-associated DMPs or saDMPs) in both our validation and discovery data sets and, from here on, are the focus of this manuscript (Additional file 6: Figure S1C). CpG sites which we identified to have higher methylation in females are from here, referred to as ‘female-biased CpGs’ and CpG sites which have higher methylation in males are here on referred to as ‘male-biased CpGs’. We found that these saDMPs were distributed across all autosomes (Fig. 1A) with 74% of the saDMPs being female-biased CpGs (293 CpGs) and 26% being male-biased CpGs (103 CpGs) (Fig. 1B) (see Additional file 1 for the full list).

Since we had such stringent parameters to define what we considered a significantly associated saDMP for males and females, we performed principal component analysis (PCA) to see how male and female beta values clustered in PC space and to evaluate the effect of DNAme at the saDMPs. As shown in Fig. 1C, male and female samples formed distinct clusters based on the beta values of the significant sex-associated DMPs (396 CpGs). PC1 explained 16.1% of the variance and PC2 explained 4.2% of the variance. Based on Fig. 1C we can conclude that these saDMPs are sufficient to contribute to the clear separation of male and female samples in PC space.

### Characterisation of sex-associated DMPs

The saDMPs were found in 174 unique genes with 48 of these genes harbouring several saDMPs (Fig. 1D). The number of saDMPs harboured by individual genes ranged from 1 to 8. CRISP2, a gene known to be involved in sperm function and male fertility [40], harboured the largest number of saDMPs, 8, which interestingly were all found to be female-biased CpGs. We performed GO and KEGG analyses but did not identify any significantly enriched biological processes or pathways for these genes. Nevertheless, the genes which did harbour saDMPs are biologically interesting, as many are genes known to be involved in sexual development and processes, such as SOX18 [41]. Further, some genes are already known to exhibit sex specific methylation patterns, such as PRR4 and PTPRN2 [42, 43]. Despite this, we were able to identify some novel genes which have not previously been reported to exhibit sex differences in DNA methylation such as GCK, HIP1R and KANK1.

To help us gain more insight into the functional role of these saDMPs, we characterised their genomic location and further compared this with the autosomal EPIC background. We found that saDMPs are preferentially located in CpG islands and CpG shores and depleted in open sea regions compared to the autosomal background (Fig. 1E). Moreover, female-biased CpGs are enriched at promoters and exons, with male-biased CpGs being enriched at 5’UTRs (Fig. 1F). Interestingly, we observed that all saDMPs display enrichment at enhancers, which, together with their presence at promoters, indicates that they could play a role in gene regulation. Lastly, we also note that all saDMPs were depleted at transposable elements and introns compared to the autosomal EPIC background.

Enrichment of saDMPs at enhancers suggests that some of the saDMPs could potentially regulate distal genes [44, 45]. We further annotated the saDMPs to genes by identifying if their contacts with promoters are mediated by 3D chromatin loops detected in Hi-C data. Following this, we further annotated the saDMPs to 37 additional genes, 28 of them being annotated to female-biased CpGs and 8 to male-biased CpGs (see Additional file 7: Figure S2A, B and Additional file 5).

Of the 8 genes linked to male-biased CpGs, we found three histones (HIST1H3A, HIST1H4A and HIST1H4B), which are known to interact with CDYL. Chromodomain Y-like protein (CDYL) is a chromatin reader binding to heterochromatin (H3K9me3, H3K27me2 and H3K27me3) that is crucial for spermatogenesis, male fertility and X chromosome inactivation [46]. In addition, ODF2L; outer dense fibre of sperm tails 2 like is linked to saDMPs female-biased CpGs and has previously been shown to interact with PRSS23, which is involved in ovulation [47].

Next, to evaluate whether the genes controlled by the saDMPs are part of the same regulatory network, we merged all proximal and distal genes and produced protein–protein interaction networks to visualise the networks of these genes. Following this, we were able to identify the top 30 hub genes by evaluating each gene by its network connectivity. The results for these analyses are produced in Additional file 7: Figure S2C, D. The top hub gene (ranked by the maximum clique centrality method) for the male-biased CpGs in males was HIST1H4B and the top hub gene for female-biased CpGs was SLC17A7.

### Enrichment of saDMPs in transcription factor binding sites

**Fig. 2:**
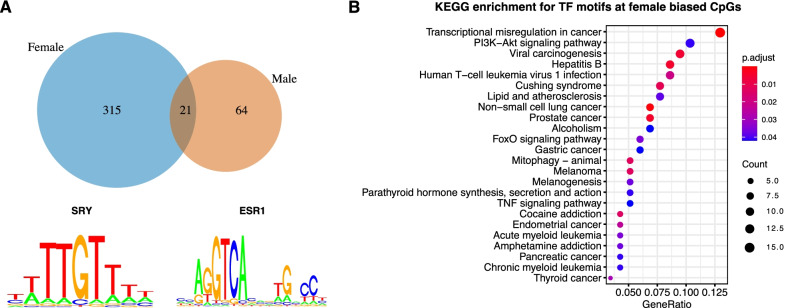
Transcription factor motif enrichment analysis. **A** Overlap of enriched TF motifs for female-biased CpGs (blue) and male-biased CpGs (orange). saDMPs were enriched in TF binding motifs including SRY and ESR1. **B** KEGG analyses for the significantly enriched TF motifs at female-biased CpGs

To identify common features among the sex-associated DMPs, we performed transcription factor (TF) binding site and gene ontology analyses. First, we evaluated whether the saDMPs were enriched in motifs for TFs (100 bp window). For the 293 female-biased CpGs, we found 315 unique enriched TFs (*p* value < 0.05) (Fig. 2A and Additional file 3) with strongest evidence for FOXB1, TIA1 and XRCC1. These are genes not previously reported to exhibit any sex differences or be enriched at areas exhibiting any sex differences. We did however find some TF motifs enriched which have previously been shown to play a role in sexual development and hormone levels. For example, we found SOX13, SOX21 and SRY TF motif to be enriched in female-biased CpGs, which are known to be involved in male sex determination (Fig. 2A) [2, 48]. For the 103 male-biased CpGs, we identified 64 enriched TFs, including ESR1 which encodes the oestrogen receptor, TCEAL6 HLCS and GPD1 (Fig. 49A and Additional file 4).

To analyse whether the TF motifs were enriched for annotation to biological processes or pathways, we performed pathway analyses using the GO and KEGG databases in order to learn more about how potential sex specific regulatory pathways affected different pathways. We identified several enriched KEGG pathways for the TFBS enriched at female-biased CpGs, spanning a wide range of processes such as transcriptional misregulation in cancer, several specific cancer pathways, PI3K-Akt signalling and more (Fig. 2B). In addition, we also found 39 enriched GO terms ranging from transcription factor activity, E-box binding, transcription coactivator activity and interestingly, bHLH transcription factor binding (Additional file 8: Figure S3B). Nevertheless, we found no enriched KEGG terms for the TFs enriched at male-biased CpGs, likely due to the small number of enriched TFs. However, we identified several enriched GO terms such as NAD, NADP binding and oxidoreductase activity (Additional file 8: Figure S3A).

As we identified enrichment for some transcription factors encoded on the sex chromosomes (e.g. SRY), we hypothesised that sex chromosome encoded transcription factors may influence CpG methylation at the saDMPs directly or indirectly by acting as hub genes in the enriched TF motif network. To assess this, we firstly produced protein–protein interaction networks to visualise the networks of these TFs (Additional file 9: Figure S4). Although we identified some enriched motifs for several TFs encoded on the X chromosomes in the male-biased CpGs such as ELK1, TGIF2LX and TCEAL6 (Additional file 9: Figure S4A), we observed that they were not central nodes in the network. Nevertheless, we did identify several central TF motifs encoded on the sex chromosomes for the female-biased CpGs (Additional file 9: Figure S4B). These included 15 TFs encoded on the X chromosome and 2 on the Y chromosome including SRY and KDM5D.

Secondly, we further utilised cytohubba a plug-in tool in cytoscape to robustly identify if these TFs were in fact hub genes in the network. This revealed that one TF encoded on the X chromosome (RPS4X) did in fact act as a hub gene in the TF network; however, the other 29 genes were encoded on the autosomes (Additional file 9: Figure S4C). Furthermore, for the TF motifs enriched at female-biased CpGs, we identified MAPK1, JUN and BRCA1 and other autosomal genes to be hub TFs in the network revealing novel TFs involved in sex differences (Additional file 9: Figure S4D). Moreover, for those TFs enriched at male-biased CpGs, we identified SP1, ESR1 and SMAD4 to be hub genes in this network (Additional file 9: Figure S4C). Interestingly, SP1 is a gene known to influence SRY expression [49] and ESR is the gene that encodes the oestrogen receptor and lastly, SMAD4, has previously been described as a female germ cell determinant [50]. This analysis suggests that although we did identify some sex chromosome encoded TFs to act as hub genes in the TF network, it is unlikely that they are responsible for affecting CpG methylation at these saDMPs.

### Relationship with gene expression

The 396 saDMPs were then further explored in association with the expression levels of their annotated genes using publicly available data for whole blood poly(A) + (GSE120312). The majority of the differentially expressed genes (DEGs) are located on the sex chromosomes, but we also did observe differential expression between males and females for several autosomal genes (Additional file 10: Figure S5B-S5C). We did not identify any significant sex-biased gene expression patterns corresponding to differences in DNAme levels at these genes (Additional file 10: Figure S5). This is not surprising as it has been previously reported that autosomal sex differences in DNA methylation result in nominal or no differences in gene expression [26, 28], a trend also seen with age specific DNAme marks [51]. Moreover, while other studies claim that they identify DEGs on autosomes between males and females, corresponding to differences in DNA methylation, when adjusting for multiple testing, it appears that these no longer hold statistical significance [27]. It is also important to note that, the relationship between DNAme with gene expression is a complex one, although it is generally thought that DNA methylation leads to gene repression, lots of literature reports methylation leading to active expression [52–54] or that it is insufficient to repress transcription [55]. These results support the idea that differences in DNA methylation observed between males and females do not lead to significant differences in gene expression.

### Sex-associated differentially methylated regions

Given that several genes harboured numerous saDMPs, we postulated whether some of the saDMPs were part of larger differentially methylated regions associated with sex. We therefore searched for differentially methylated regions associated with sex in our discovery and validation data set. Following adjustment for multiple testing (FDR) and adjustment for cell type proportions, batch effects and age, we identified many sex-associated differentially methylated regions. We therefore considered a sex associated differentially methylated regions (saDMRs) as significant if it harboured at least 5 CpGs, had an FDR value smaller than 0.05, had a methylation difference within the region greater than 0.05 in either direction and was present in both our discovery and validation data set. Following filtering of the list of saDMRs, we identified 266 significant sex-associated DMRs on the autosomes between males and females located at 231 unique sets of genes (Additional file 2). The number of CpGs within the DMRs ranged from 6 to 123 and had an average width of 2392 base pairs (bp) ranging from 178 to 14,715 bp.

**Fig. 3:**
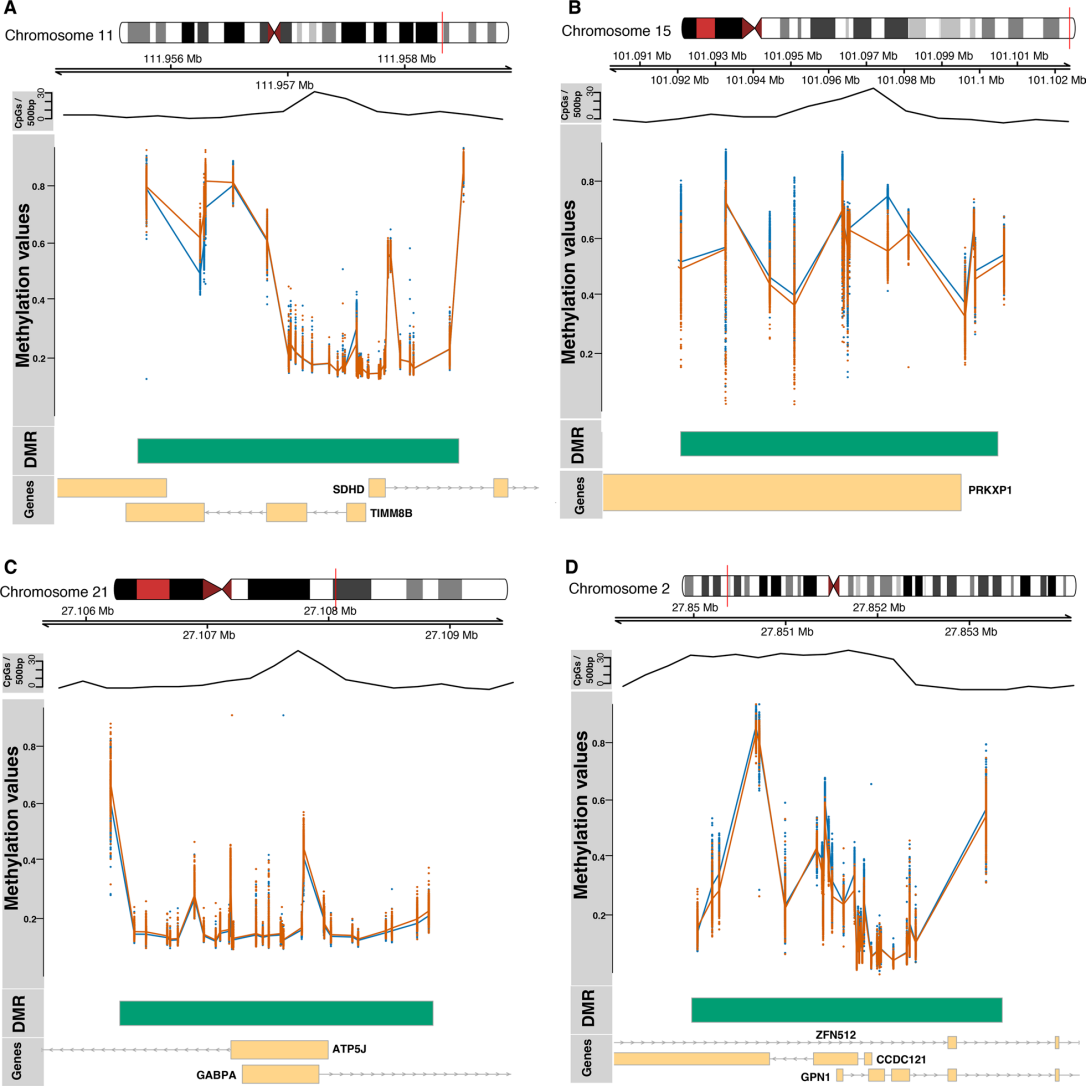
Plots of sex-associated differentially methylated regions (saDMR). We plotted regions: **A** SDHD and TIMM8B, **B** PRKXP1, **C** ATP5J and GABPA and **D** CCDC121 and GPN1. Yellow boxes represent appropriately labelled genes, green boxes represent the genomic region which the differentially methylated region spans. The scatterplots represent the beta values for males (orange) and females (blue) at CpG sites located within the differentially methylated region

Figure 3 shows the beta values for males and females at 4 of the most significant saDMRs: The top hits in the saDMR list overlapped promoter regions of genes such as SDHD, TIMM8B, ATP5J, GABPA, GPN1, CCDC121, AND PRKXP1. SDHD and TIMM8B are genes known to be influenced by oestrogen exposure [56] suggesting that sex hormones may underlie sex differences in autosomal DNA methylation, or alternatively that DNA methylation may mediate sex hormone levels. Moreover, ATP5J and GABPA are genes (male-biased CpGs) which have previously been reported to be implicated in early onset of Alzheimer’s disease [57, 58], a disease known to affect females more than males. Furthermore, ATP5J is a gene known to be a target gene of oestrogen, previously shown to serve an inhibitory role in the sex differences in hepatocellular carcinoma [59]. GPN1, CCDC121, ATP5J and GABPA have previously been shown to exhibit functions which are sex specific [21]. Furthermore, PRKXP1 is located on chromosome 15 and CpGs in this region have previously been associated with Crohn's disease and intestinal inflammation, a disease which has previously been reported to be more prevalent in females [60]. A saDMR harbouring 123 CpGs overlapped the promoter region of a gene called MCDC1, a gene known to direct chromosome wide silencing of the sex chromosomes in male germ cells, initiate meiotic sex chromosome inactivation (MSCI), and lead to XY body formation [61].

These findings are extremely important for epigenome wide association studies aiming to characterise sex specific effects in relation to exposures, a rising theme in the literature [6, 42, 62–65]. Our study provides a valuable resource for the community to disentangle whether particular sites or regions display sex differences in DNA methylation.

## Discussion

Here, we conducted the first study aiming to characterise autosomal sex differences in DNAme between males and females in whole blood using the Illumina EPIC BeadChip, which interrogates ~ 850,000 sites across the genome. While we were able to identify thousands of autosomal CpGs displaying sex differences in DNA methylation, we focused the majority of our analysis on those autosomal CpGs displaying the largest sex differences (see Methods). We, thereby, identified 396 sex-associated differentially methylated positions on the autosomes. Previous work has reported contradicting results, some research report that there is higher methylation on autosomes in females [5, 19, 21], while other research reports identifying higher methylation on autosomes in males [28–66] and others reports no significant difference in DNAme on autosomes between males and females [68]. Our results support the former and we found that 76% of these loci (293 CpGs) showed higher methylation in females compared to males.

Therefore, although the existence of these autosomal sex-associated CpG sites is well established, a robust and consistent catalogue is yet to emerge. When comparing the saDMPs discovered in this study to findings previously reported in blood samples (where a full list of sex-associated sites were available), we do observe some overlap, although it is limited (Table 2). For example, we identify 54% overlap of our identified saDMPs with previously reported sex-associated sites in cord blood [21]. On the other hand, overlap from other research also investigating cord blood was lower, namely 36.87% [69]. Furthermore, we observe only ~ 15% overlap of saDMPs identified in a previous study investigating sex differences in peripheral leukocytes [24]. We also additionally checked the overlap between our saDMPs identified in blood with those identified in other tissues (Table 2). Interestingly, we observe a 73% overlap with sex-associated sites identified in placenta by Inkster et al. [27] and a 35% overlap with sex-associated sites identified in post-mortem prefrontal cortex [66]. The existence of this overlap between different tissues shows that a portion of these saDMPs identified are conserved across tissues, and that some are tissue specific. This highlights an important avenue for future work.

**Table 2:** Overlap of autosomal sex-associated differentially methylated positions reported in this study with previous literature in various tissues

Study	Tissue of interest	Sample size (number of individual samples in study)	Platform used for DNA methylation assessment	Number of autosomal probes identified	Percentage of saDMPs replicated (%)	References
Yousefi et al. (2014)	Cord blood	111 newborns	Illumina 450 k	3031	54	[21]
Mccarthy et al. (2014)	Meta analysis of 76 studies	6795	Illumina 27 k	184	0.54	[70]
Inoshita et al. (2015)	Peripheral leukocytes	117 adults	Illumina 450 k	292	15	[24]
Maschietto et al. (2015)	Cord blood	71 newborns	Illumina 450 k	2332	36	[69]
Xu et al. (2014)	Post-mortem prefrontal cortex	46 adults	Illumina 450 k	614	35	[66]
Hall et al. (2014)	Pancreas	87 adults	Illumina 450 k	470	18	[19]
Xia et al. (2021)	Post-mortem brain samples	1408 adults	Illumina 450 k	15,417	31	[7]
Inkster et al. (2021)	Placenta	293 adults	Illumina 450 k	162	73	[27]

However, alternative reasons for this limited overlap exist; firstly, this may be attributed to differences in sample size, as the datasets used within this study are much larger than previously used thus increasing our ability to detect true sex differences in DNA methylation.

Secondly, the differing normalisation methods applied to DNAme microarray data. Previous research has shown that the methylation levels of CpG sites on the X chromosome differ largely between males and females [29] and, thus, normalisation methods which normalise array data indiscriminately with CpG sites on the autosomes introduce large technical biases for autosomal CpGs [30]. Using normalisation methods, which do not handle the technical bias introduced by sex chromosomes, will therefore lead to many autosomal CpG sites being falsely associated with sex and further, a higher number of autosomal CpGs being incorrectly identified as male-biased CpGs. Our choice of normalisation method greatly reduced technical bias at autosomal CpGs for male and female samples.

Moreover, as thousands of autosomal CpG sites show differences in DNA methylation between males and females, differences in the methods for determining the definition of a sex-associated site result in limited reproducibility between studies, a point also raised by Gatev and colleagues in their identification of sex-associated regions [28]. Here, we therefore proposed and applied stringent cut offs to define a sex-associated site (FDR < 0.05 and effect size of at least 0.05 in either direction). While we acknowledge that true but small differences in DNA methylation related to a phenotype may exist, in the interest of generating a reproducible and robust catalogue of saDMPs, we chose to apply effect size cut offs. Consistent with this, we were able to replicate 75% of our saDMPs identified in our validation data set in our discovery data set. Moreover, we found that 73% of the saDMPs we identified in this study were also identified by Inkster and colleagues [27], whom also applied effect size cut offs, demonstrating the reproducibility and robustness of our catalogue of saDMPs.

We further categorised these 396 saDMPs into two groups, those that were male-biased CpGs (*n* = 103) and those that were female-biased CpGs (*n* = 293). Several saDMPs found to be female-biased CpGs overlapped the transcription start site (TSS) of genes not previously been reported to exhibit sex differences in DNAme including C19orf77, ATP10D and SHANK2. Interestingly, it has previously been shown that sex hormones can regulate SHANK expression leading to a sex differential expression in SHANK2 [71]. Furthermore, this gene has previously been implicated in autism spectrum disorder, a disorder known to exhibit higher prevalence in males rather than females [21]. In contrast, the most significant male-biased CpG is located in the CpG island of a gene located on chromosome 21 called GABPA. GABP is a methylation sensitive transcription factor and has previously been shown to be a transcriptional activator of Cyp 2d-9, which is a gene encoding a male specific steroid in mice [72]. Sex differences in these regions have previously been identified by other studies investigating autosomal sex differences in DNA methylation, specifically Yousefi et al. [73] also identified this region to be a top sex-associated DMR in their analysis. In addition, previous research investigating transcriptome wide sex differences using single cell RNA-seq data in mouse reports GABPA to be one of six TF families responsible for the majority of sex dimorphic transcriptional regulation activities [74].

Interestingly, as well as GABPA being the gene annotated to the most significant saDMP (male-biased CpGs), it was also the third most significant saDMR, suggesting these regions could account for important sex biases observed in some diseases. This is further supported by the fact that GABPA has also been heavily associated with early onset of Alzheimer's disease, Parkinson's disease, breast cancer and autism [71, 73–75].

The saDMR harbouring the highest number of CpG sites (*n* = 123) is located on chromosome 6, overlaps TUBB, MDC1 and XXbac. MDC1 is thought to play a crucial role in the production of male games, lead to XY body formation and also initiate meiotic sex chromosome inactivation. These functions are achieved through its interaction with DNA damage response (DDR) factors, ultimately leading to transcriptional silencing [61, 76].

These results collectively support the hypothesis that sex differences in autosomal DNAme may account for some of the sex differences seen in disease prevalence, onset and progression. Moreover, we did identify saDMPs in genes known to exhibit sex differences in DNAme such as CRIPS2 and DDX43 which are involved in spermatogenesis and male fertility [21, 40], Specifically, CRIPS2 harboured 8 significant saDMPs, all female-biased CpGs, and is part of a group of proteins called CRISPs which show male-biased expression in the male reproductive tract. CRIPS2 plays an important role in spermatogenesis, acrosome reaction and gamete fusion [40]. Some of our saDMPs were located in genes known to show sex by age effects, such as PRR4, a gene associated with dry eye syndrome [77]. Despite this, recent research shows that the adult blood DNA methylome is largely affected by sex, but that these methylome sex differences do not change throughout adulthood and so are largely independent from age effects [78].

The Illumina EPIC array has an increased coverage of the genome, including distal regulatory elements [79]. It was interesting that the 396 saDMPs were still found to be significantly enriched at CpG islands and CpG shores but depleted in open sea regions of the genome (Fig. 1E). The genomic location of DNA methylation normally alters its function. Methylation in CpG islands normally functions to serve long term silencing of genes [80] and CpG island shore methylation is strongly related to gene expression [81], suggesting a potential functional role for these saDMPs. To further support these findings, we identified enrichment of these saDMPs at enhancers, 5’UTRs and promoters (Fig. 1F). Enrichment at 5’UTRs is potentially suggestive that they may be acting as alternative promoters, though we did not test this hypothesis in this study. Despite this enrichment at regulatory regions, we found no correlation of these sites alone with significant differences in gene expression between males and females, suggesting that these saDMPs are not sufficient alone to predict gene expression. Further, this suggests that DNA methylation may potentially be acting as a passive reporter of sex specific transcription. Moreover, it is well established that DNA methylation differences do not always result in differences in gene expression but that these DNA methylation differences are likely to instead be part of larger gene regulatory networks, via acting distally or interacting with transcription factors [52, 53, 82–84]. Despite this, we acknowledge that one caveat of our study was that our DNA methylation data and gene expression data were obtained from different cohorts (they are unmatched) and have large differences in sample size (RNA-seq data have a significantly smaller sample size).

However, this potential link was identified in our TF motif analysis, where we found SRY (sex determining region Y) transcription factor motif, also known as the sex determining factor, to be enriched at female-biased CpGs and further identified this gene to be acting as a hub in the TF network. SRY has been found to bind and repress WNT activation of ovarian genes, and has been shown to bind the promoter regions of many targets of involved in differentiation of the testis [48, 49, 85]. Furthermore, we also found ESR1 transcription factor motif enriched in the saDMPs female-biased CpGs, a gene known to code for the oestrogen receptor.

It has previously been reported that 3D genome organisation can impact sex-biased gene expression through direct and indirect effects of cohesion and CTCF looping on enhancer interactions with sex-biased genes [10]. Recently, it was shown that with rising oestrogen levels, the female brain exhibits sex hormone driven plasticity and that chromatin changes underlie this [86]. Interestingly, by annotating our saDMPs to distal genes using chromatin loops, we were able to identify contacts between saDMPs and three genes HIST1H3A, HIST1H4A and HIST1H4B which are core components of nucleosome, thereby responsible for playing a role in chromatin organisation. Note that the Hi-C data and DNA methylation data were not from matched samples, but two different cohorts. However, these results suggest that although we found DNAme to not be predictive of sex differences in gene expression (Additional file 87: Figure S5), these saDMPs may interact with other genes, transcription factors and other epigenetic modifications to direct chromatin organisation and regulatory networks.

Lastly, we acknowledge limited overlap with previous studies yet conclude that this is due to our extremely large sample size (discovery, *n* = 1171 and validation, *n* = 2471) and improved handling of sex bias introduced by normalising such data with the sex chromosomes. Both factors contribute to our ability to detect true positives and obtain a more robust catalogue of true sex-associated autosomal CpGs.

## Material and methods

### Participants

Whole blood Illumina Infinium MethylationEPIC BeadChip DNAme data were collected from participants involved in Understanding Society: The UK Household Longitudinal Study [31]. In wave 3 of the study (2011–12), blood samples were collected from a portion of the study participants. Individuals were considered eligible to give a blood sample if they were over the age of 16, consented to blood sampling and genetic analysis, and participated in all annual interviews between 1999 and 2011 as previously reported in Hughes et al. [88]. Our study population was restricted to participants of white ethnicity. A full description of the dataset and data processing has been described by [88]. Following quality checks of the data, our final data set consisted of 1171 participants (males = 489, females = 686) for discovery and 2471 (males = 1135, females = 1345) participants for validation. The age ranges for each data set were 28–98 years old and 16–99 years old, respectively.

### DNA methylation data

Samples of whole blood DNA from participants were obtained following the protocol described in [88]. Raw signal intensities were processed using the R package bigmelon [29] and watermelon [89] from idat files. Prior to normalisation of the data, outlier samples were identified using principal component analysis and subsequently removed from the data set. The reported age of each sample was compared to predicted age using the epigenetic age method implemented by *agep* in the R package bigmelon [89]. Further, the reported sex of the samples was checked using a DNA methylation-based sex classifier [90] which predicts sex based on the methylation difference of X and Y chromosomes. 4 samples were subsequently removed from our discovery data set and 9 samples were removed from our validation data set, as reported and predicted sex did not match. The data were then normalised via the *interpolatedXY* adjusted *dasen* method implemented in the R package, wateRmelon [91]. Following normalisation of the data, SNP probes, cross hybridising probes 27 and X or Y linked probes were removed from the data set. The final discovery and validation data set consisted of 1171 and 2471 samples, respectively, and 747,302 DNA methylation sites.

As whole blood is a heterogenous tissue and contains different cell types, individual samples will have different cell type proportions which may confound analyses. Often, this manifests itself as many false positives being identified. The estimation is based on epigenetic data and expected DNA methylation signatures at specific loci in each cell types are used to estimate cell type composition. To ensure that whole blood cell composition did not differ significantly by sex and would not introduce bias to our results, the relative proportions of Granulocytes, mononuclear, natural killer, CD4T, CD8T and B cells were estimated for all samples using the *estimateCellCounts* function implemented in bigmelon [89]. Furthermore, to assess whether the sex differences we observed were age independent, we performed a Mann–Whitney U test between the age distribution of males and females. Our results confirmed that there is no statistical difference in age between our male and female samples for our discovery data set (*p* value 0.07; median values of 60 and 58, respectively) and also for our validation data set (*p* value 0.26; median values of 52 and 51, respectively).

### Identifying sex-associated autosomal differential methylation.

Sex-associated autosomal differentially methylated positions (saDMPs) were identified by performing linear modelling using the limma package in R [92] using sex annotation and Beta values while adjusting for age, cell type proportions and batch effects. Correction for multiple testing was performed with the Benjamini–Hochberg false discovery rate method (FDR values). We further used the Bayesian method for controlling p value inflation using the R package *bacon* for both our discovery and validation data sets [93]. A probe was considered significantly differentially methylated if the difference in Beta values between males and females was greater than 0.05 in either direction and the FDR value was smaller than 0.05. We considered a saDMP to be validated if it met these two criteria in both the discovery and validation data set. We further characterised differentially methylated regions (DMRs) by applying the *DMRcate* function from the R package ChAMP to detect DMRs between males and females on the autosomes [94]. A DMR was considered to be significantly associated with sex (saDMR) if it consisted of at least 5 CpG sites with a maximum difference in beta values between males and females greater than 0.05.

### Genomic annotation of CpG sites

We annotated the autosomal CpG’s using the manufacturer supplied annotation data (MethylationEPIC_v-1-0_B2 manifest file). Annotation was completed in the R package Minfi [95]. Several categories were used as annotations in relation to CpG islands and divided into the following categories: CGIs, CGI shores (S and N), CGI shelfs (S and N) and open sea regions. Further, we also annotated the autosomal CpGs to several genomic features, including exons, introns, 5’ UTR, 3’UTR, enhancers, promoters and transposable elements (TEs) using data from UCSC table browser (https://genome.ucsc.edu/cgi-bin/hgTables).

### Gene ontology analyses

GO analyses were conducted using the *gometh* function in the missMethyl package [96] which tests gene ontology enrichment for significant CpGs while accounting for the differing number of probes per gene present on the EPIC array. For GO ontology analyses of enriched TFBS, we used *enrichGO* from the clusterProfiler package in R [97], which performs FDR adjustment .

### Enrichment of saDMPs in transcription factor binding motifs and integration with gene expression

The enrichment analysis of known motifs in sex-associated DMPs was performed using the R package PWMEnrich [98] using the MotifDb collection of TF motifs [99]. Specifically, the DNA sequences within a 100 bp range from the saDMP which were female-biased CpGs were extracted from the genome and compared to the saDMPs which were male-biased CpGs as the background to reveal unique enriched motifs (adjusted *p* value < 0.05). RNA-seq data for 20 healthy donors (10 males and 10 females) from publicly available data from GEO (GSE120312) were used in our analysis. We used the pre-processed count matrices with DESeq2 [100] to calculate differentially expressed genes between males and females with an adjusted *p* value of 0.05 and log_2_ fold change of 1. DESeq2 does apply an automatic filtering step to remove genes with low counts but we did also apply our own independent filtering to this data by removing genes that have counts of at least 10 in all samples.

### Overlap of saDMP’s with chromatin loops

We examined whether any of the sex-associated DMPs made 3D contacts with distal genes using Hi-C data available from the GEO under accession number (GSE124974) for white blood cells and neutrophils. Hi-C library preparation was performed using the Arima-HiC kit and pre-processing of the data was performed using Juicer command line tools [101]. Reads were aligned to the human (hg38) genome using BWA-mem [97] and then pre-processed using the Juicer pre-processing pipeline. We called chromatin loops using the HICCUPS tool from Juicer using a 10 Kb resolution. We then constructed GenomicInteractions objects to annotate saDMPs to loop anchors using the *findOverlaps* function from the GenomicRanges package using a *maxgap* of 10,000. Following this, we then annotated the corresponding anchor to the relevant gene ID. These steps then allowed us to perform network analysis in Cytoscape [102] and GO and KEGG analyses in clusterProfiler [103].

### Protein–protein network visualisation and hub gene identification

We searched all of the genes annotated to our saDMPs using the Search Tool for the Retrieval of Interacting Genes (STRING) (https:://string-db.org) database to generate our networks. We extracted protein–protein interactions with a combined score of > 0.4. Following this, we utilised the cytoscape plugin tool Cytohubba [104] in order to identify hub genes within the networks. This was done by employing the local based method called maximum clique centrality (MCC). The same analysis was applied to the enriched transcription factors found at saDMPs.

## Supplementary Information

**Additional file 1**. Significant sex-associated autosomal DMPs. Illumina Manifest annotations for all 396 significant CpG sites associated with sex on the autosomes.

**Additional file 2**. Significant sex-associated autosomal DMRs. Test results for all significant DMR’s associated with sex on the autosomes ordered by FDR value.

**Additional file 3**. Enrichment statistics for the TF motifs enriched at female-biased CpGs.

**Additional file 4**. Enrichment statistics for the TF motifs enriched at male-biased CpGs.

**Additional file 5**. Genes annotated to saDMPs via Hi-C analysis.

**Additional file 6**. **Figure S1**: (A) QQ plot and lambda values (discovery data) distribution of the adjusted p values against the null distribution for EWAS of sex in the understanding society cohort. Genomic inflation lambda score is indicated in the QQ plot to indicate statistical inflation of p values. (B) Boxplots of estimated whole blood cell type proportions for males (orange) and females (blue) in the discovery data set, estimated using the estimateCellCounts function from bigmelon. We performed a Mann-Whitney U test (p value: n.s. 0.05, *p value < 0.05, **< 0.01 and ***< 0.001). (C) QQ plot and lambda values (validation data) distribution of the adjusted p values against the null distribution for EWAS of sex in the understanding society cohort. Genomic inflation lambda score is indicated in the QQ plot to indicate statistical inflation of p values. (D) Boxplots of estimated whole blood cell type proportions for males (orange) and females (blue) in the validation data set, estimated using the estimateCellCounts function from bigmelon. We performed a Mann–Whitney U test (p value: n.s. 0.05, *p value < 0.05, **< 0.01 and ***< 0.001). (E) Venn diagram showing overlap of differentially methylated positions identified in our validation and discovery data set before and after filtering of the list of saDMPs.

**Additional file 7**. **Figure S2**: (A) Integrated genomics viewer track of chromatin loop on chromosome 6 showing two male-biased CpGs contacting H1/H4/H3/H2V/H2A. (B) Integrated genomics viewer track of chromatin loop on chromosome 1 showing a female-biased CpG contacting the ODF2L gene. Blue lines represent the chromatin loops, with black lines showing the loop anchors. Orange vertical lines represent the male-biased CpGs and blue vertical lines represent the female-biased CpGs. Purple annotations represent genes. (C-D) Subnetworks of the top 30 genes annotated to male-biased CpGs (C) and females (D). Node colour represents the degree of connectivity. The scale from red to yellow represents the top 30 enriched genes rank from 1-30, with red indicating highest degree and yellow indicating lowest degree.

**Additional file 8**. **Figure S3**: GO terms overrepresented for the significantly enriched TF motifs at male-biased CpGs (A) and female-biased CpGs (B).

**Additional file 9**. **Figure S4**: (A-B) Network visualisation of protein-protein interactions for all transcription factor motifs found to be enriched at male-biased CpGs (A) and female-biased CpGs (B). Grey coloured boxes represent individual TFs located on autosomes, while purple-coloured boxes represent TFs encoded on the X chromosome and green coloured boxes represent TFs encoded for on the Y chromosome. Grey lines represent edges between transcription factors within the protein-protein network. (C-D) Subnetworks of the top 30 enriched TF motifs at male-biased CpGs (C) and females (D). Node colour represents the degree of connectivity. The scale from red to yellow represents the top 30 enriched TF motif rank from 1-30, with red indicating highest degree and yellow indicating lowest degree.

**Additional file 10**. **Figure S5**: Volcano plot showing differential gene expression between males and females. We considered the case of: (A) genes annotated to the saDMPs, (B) sex chromosome linked genes and (C) autosomal genes. Points coloured in grey represent non differentially expressed genes. Green points represent genes which had a log2 Fold Change value greater than 1. Blue points represent genes which met the adjusted p value threshold (FDR <0.05). Points coloured in red represent genes which showed differential expression between males and females (adjusted p value<0.05 & log2FC > 1).

## Data Availability

The code to perform this analysis is available on GitHub https://github.com/livygrant97/ASD_DNAme. RNA-seq data used in the differential gene expression analysis is publicly available on GEO (Gene expression Omnibus) under the accession number GSE120312.
