# Supplementary figures and images for: Characterising sex differences of autosomal DNA methylation in whole blood using the Illumina EPIC array
Source: Clin Epigenetics. 2022 May 14;14:62. doi: 10.1186/s13148-022-01279-7 (PMC9107695; doi:10.1186/s13148-022-01279-7)

**A**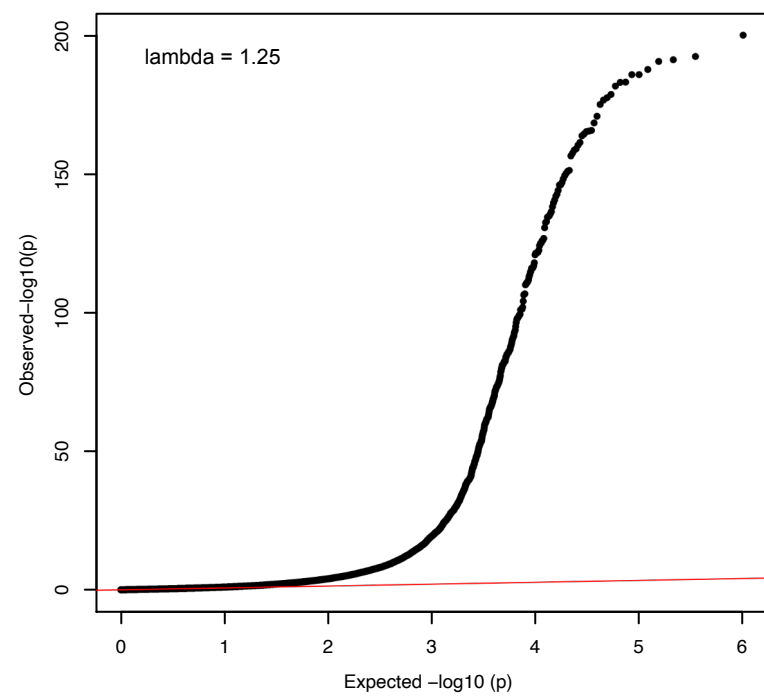**B**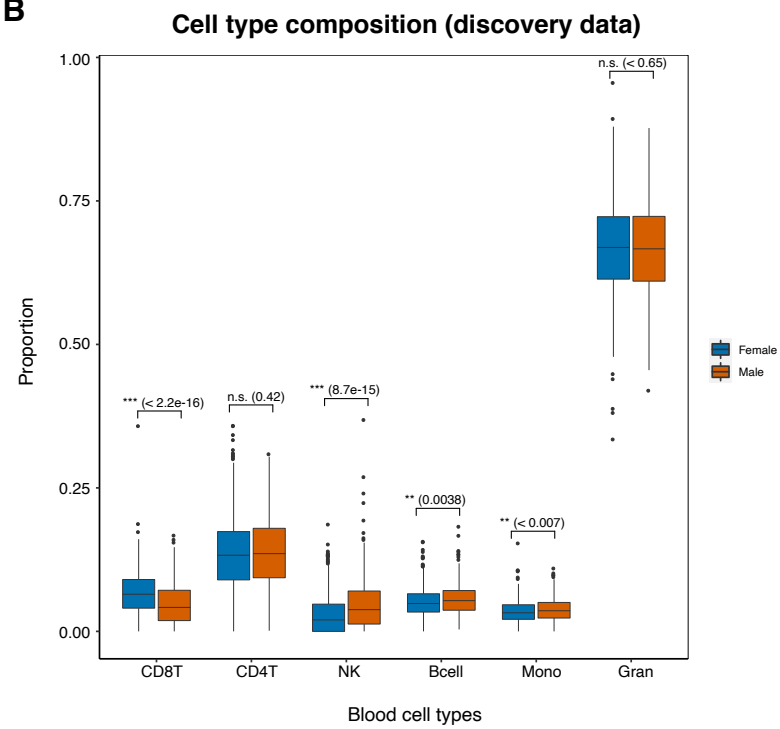**C**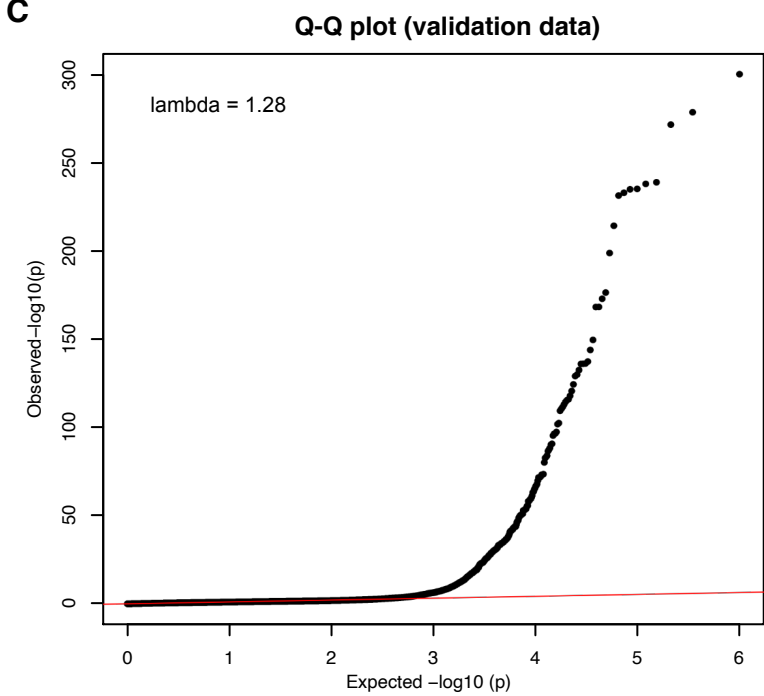**D**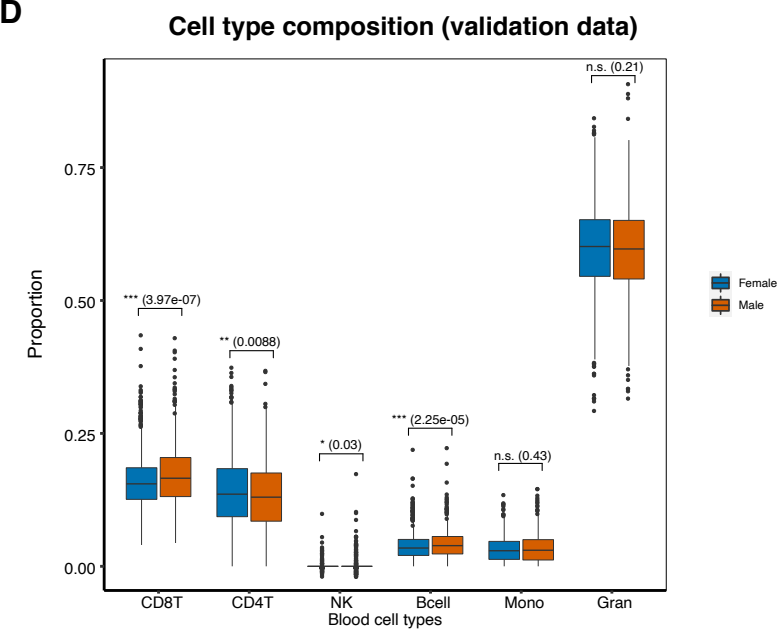**E**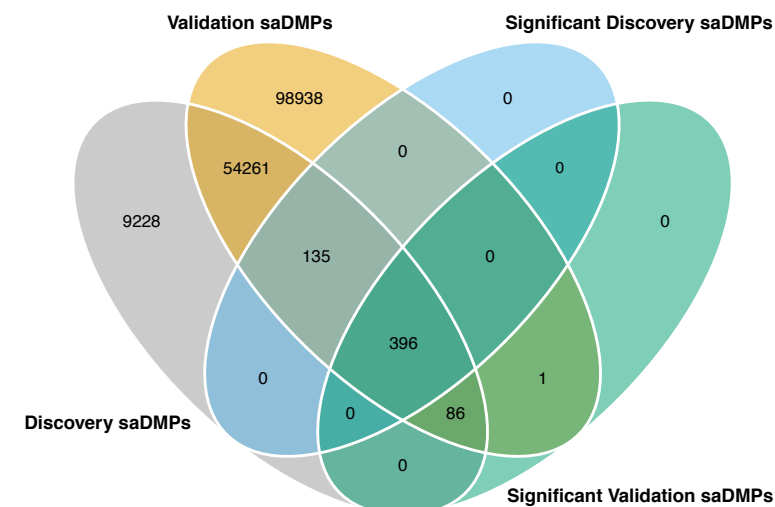

Supplement: Supplementary file 6 — Additional file 6. Figure S1: (A) QQ plot and lambda values (discovery data) distribution of the adjusted p values against the null distribution for EWAS of sex in the understanding society cohort. Genomic inflation lambda score is indicated in the QQ plot to indicate statistical inflation of p values. (B) Boxplots of estimated whole blood cell type proportions for males (orange) and females (blue) in the discovery data set, estimated using the estimateCellCounts function from bigmelon. We performed a Mann-Whitney U test (p value: n.s. 0.05, *p value < 0.05, **< 0.01 and ***< 0.001). (C) QQ plot and lambda values (validation data) distribution of the adjusted p values against the null distribution for EWAS of sex in the understanding society cohort. Genomic inflation lambda score is indicated in the QQ plot to indicate statistical inflation of p values. (D) Boxplots of estimated whole blood cell type proportions for males (orange) and females (blue) in the validation data set, estimated using the estimateCellCounts function from bigmelon. We performed a Mann–Whitney U test (p value: n.s. 0.05, *p value < 0.05, **< 0.01 and ***< 0.001). (E) Venn diagram showing overlap of differentially methylated positions identified in our validation and discovery data set before and after filtering of the list of saDMPs. [file 13148_2022_1279_MOESM6_ESM.pdf]

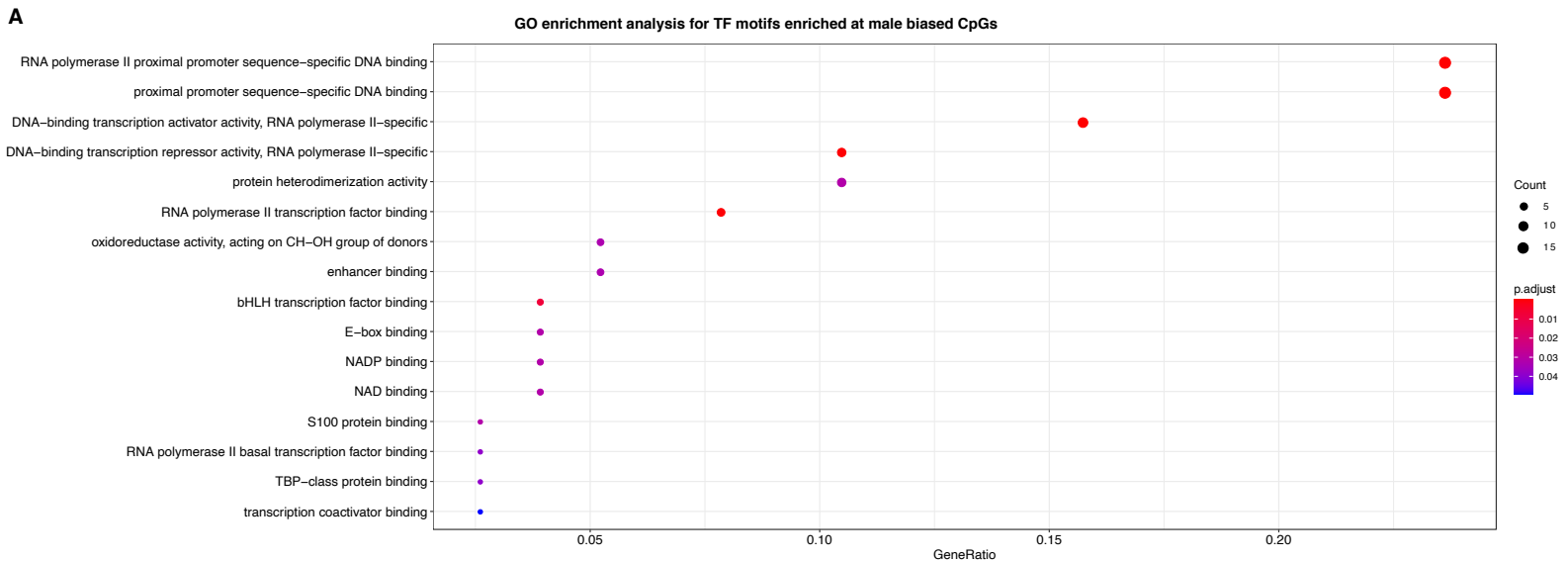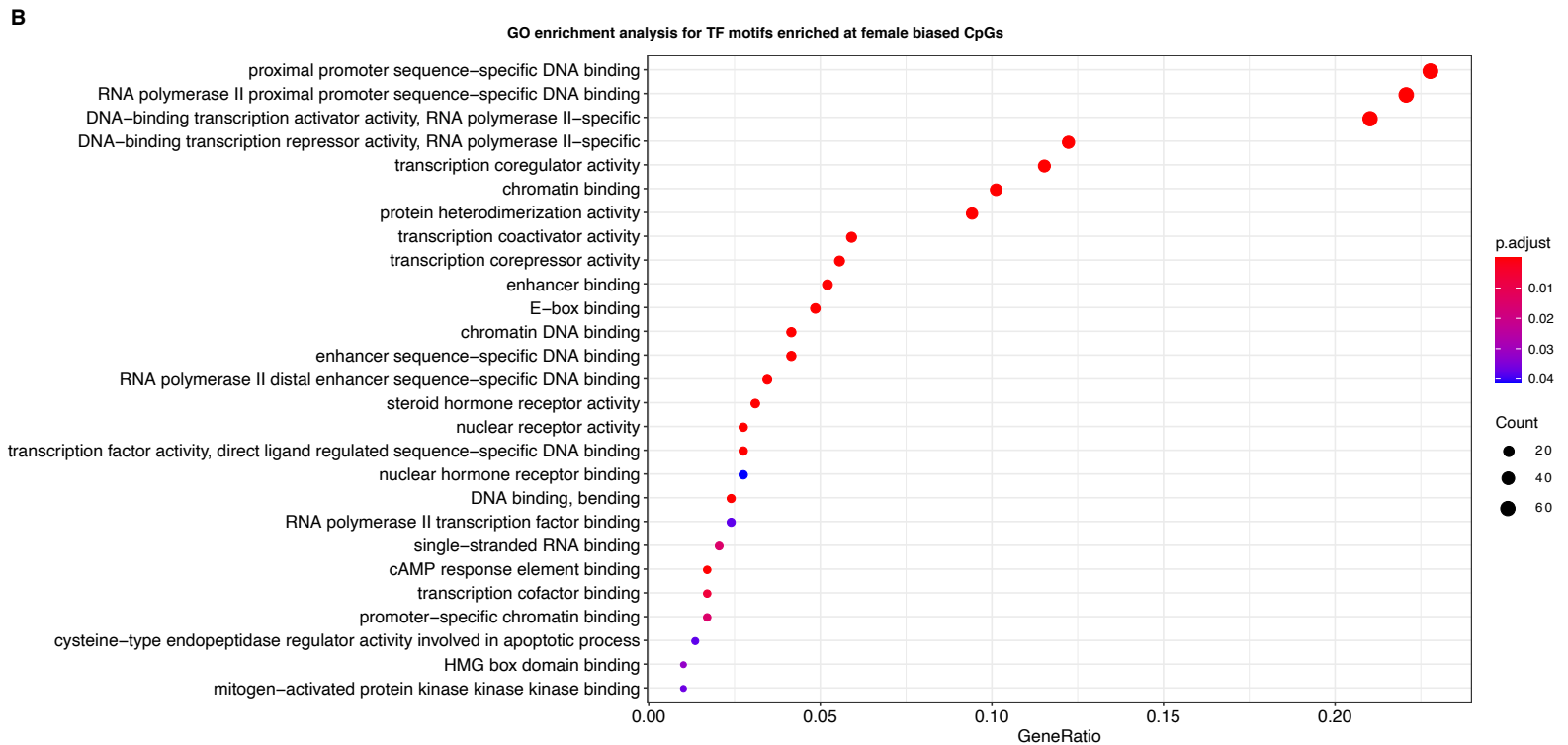

Supplement: Supplementary file 8 — Additional file 8. Figure S3: GO terms overrepresented for the significantly enriched TF motifs at male-biased CpGs (A) and female-biased CpGs (B). [file 13148_2022_1279_MOESM8_ESM.pdf]

# A

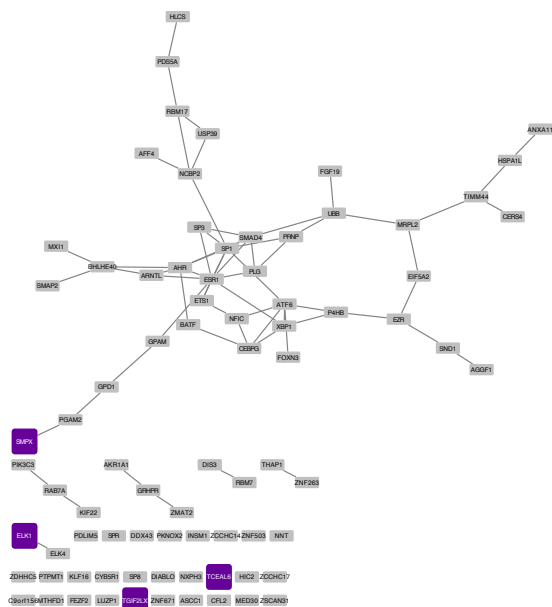

## B

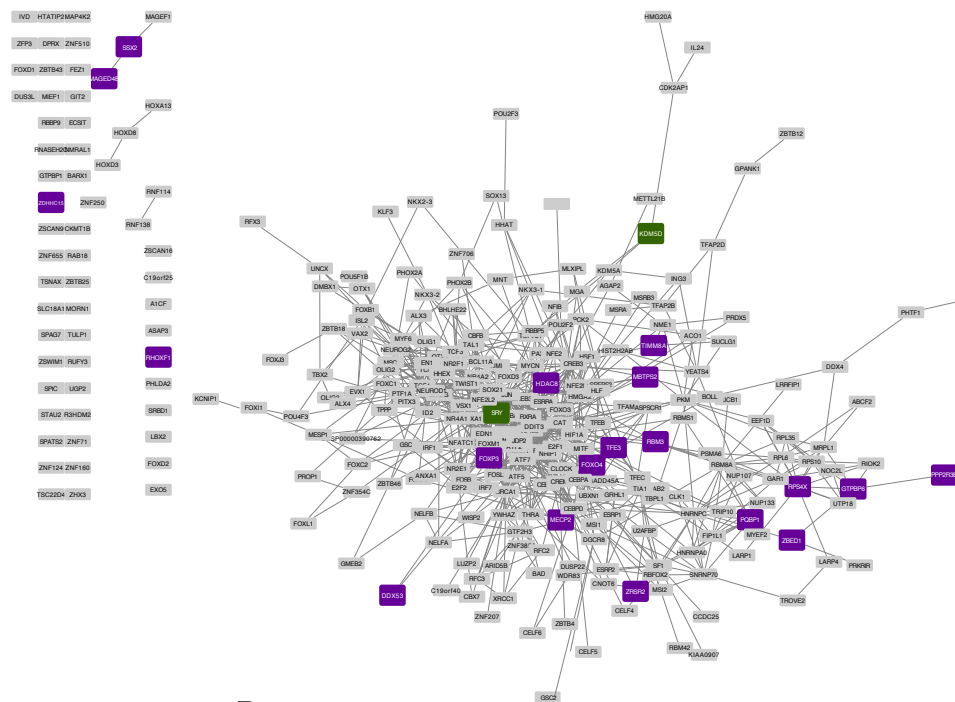

**C**

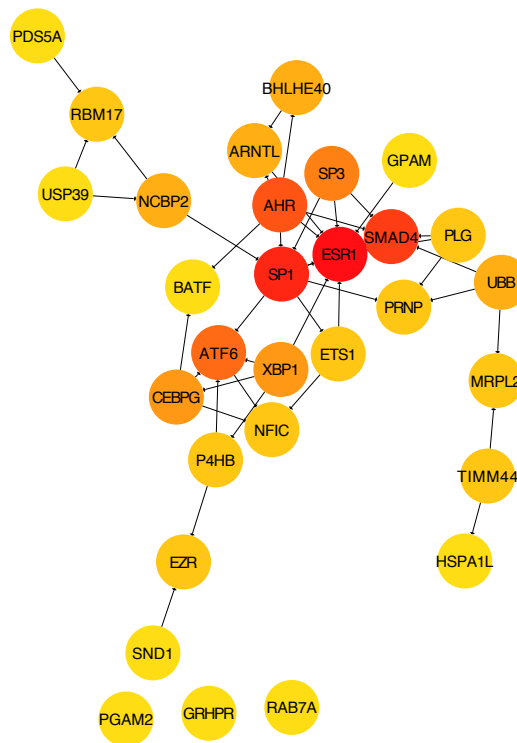

## D

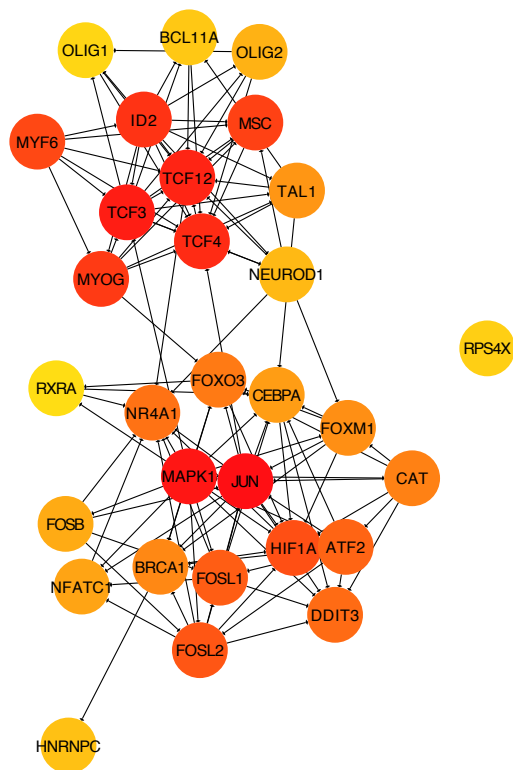

Supplement: Supplementary file 9 — Additional file 9. Figure S4: (A-B) Network visualisation of protein-protein interactions for all transcription factor motifs found to be enriched at male-biased CpGs (A) and female-biased CpGs (B). Grey coloured boxes represent individual TFs located on autosomes, while purple-coloured boxes represent TFs encoded on the X chromosome and green coloured boxes represent TFs encoded for on the Y chromosome. Grey lines represent edges between transcription factors within the protein-protein network. (C-D) Subnetworks of the top 30 enriched TF motifs at male-biased CpGs (C) and females (D). Node colour represents the degree of connectivity. The scale from red to yellow represents the top 30 enriched TF motif rank from 1-30, with red indicating highest degree and yellow indicating lowest degree. [file 13148_2022_1279_MOESM9_ESM.pdf]

**A****saDMPs**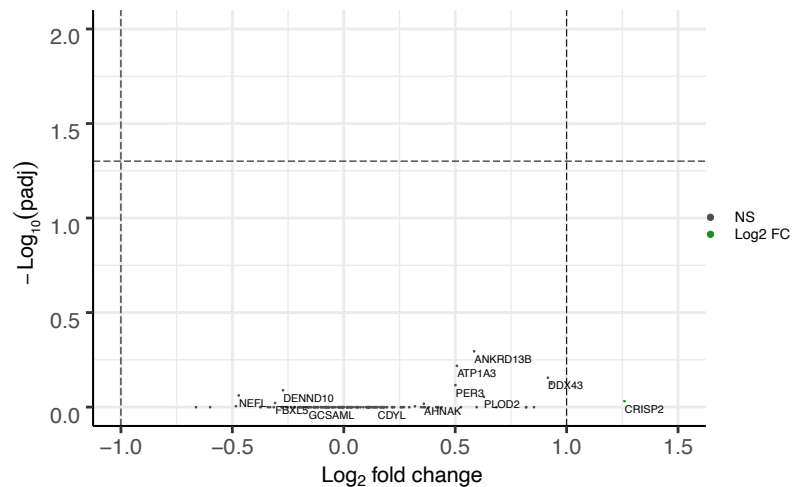**B****sex chromosomes**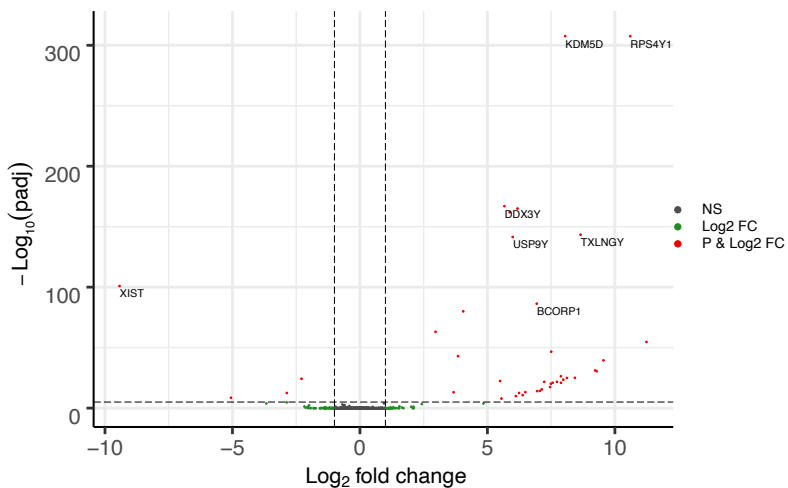**C****autosomes**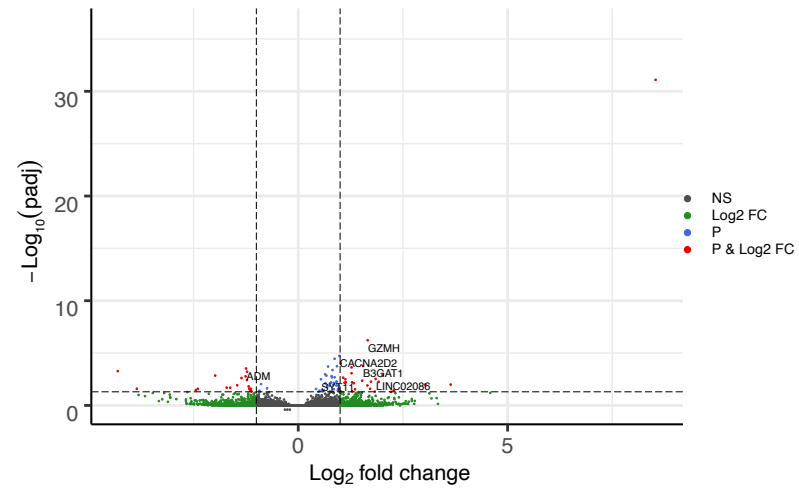

Supplement: Supplementary file 10 — Additional file 10. Figure S5: Volcano plot showing differential gene expression between males and females. We considered the case of: (A) genes annotated to the saDMPs, (B) sex chromosome linked genes and (C) autosomal genes. Points coloured in grey represent non differentially expressed genes. Green points represent genes which had a log2 Fold Change value greater than 1. Blue points represent genes which met the adjusted p value threshold (FDR <0.05). Points coloured in red represent genes which showed differential expression between males and females (adjusted p value<0.05 & log2FC > 1). [file 13148_2022_1279_MOESM10_ESM.pdf]
