# Supplementary material for: Characterising sex differences of autosomal DNA methylation in whole blood using the Illumina EPIC array
Source: Clin Epigenetics. 2022 May 14;14:62. doi: 10.1186/s13148-022-01279-7 (PMC9107695; doi:10.1186/s13148-022-01279-7)
Supplement: Supplementary file 7 — Additional file 7. Figure S2: (A) Integrated genomics viewer track of chromatin loop on chromosome 6 showing two male-biased CpGs contacting H1/H4/H3/H2V/H2A. (B) Integrated genomics viewer track of chromatin loop on chromosome 1 showing a female-biased CpG contacting the ODF2L gene. Blue lines represent the chromatin loops, with black lines showing the loop anchors. Orange vertical lines represent the male-biased CpGs and blue vertical lines represent the female-biased CpGs. Purple annotations represent genes. (C-D) Subnetworks of the top 30 genes annotated to male-biased CpGs (C) and females (D). Node colour represents the degree of connectivity. The scale from red to yellow represents the top 30 enriched genes rank from 1-30, with red indicating highest degree and yellow indicating lowest degree. [file 13148_2022_1279_MOESM7_ESM.pdf]

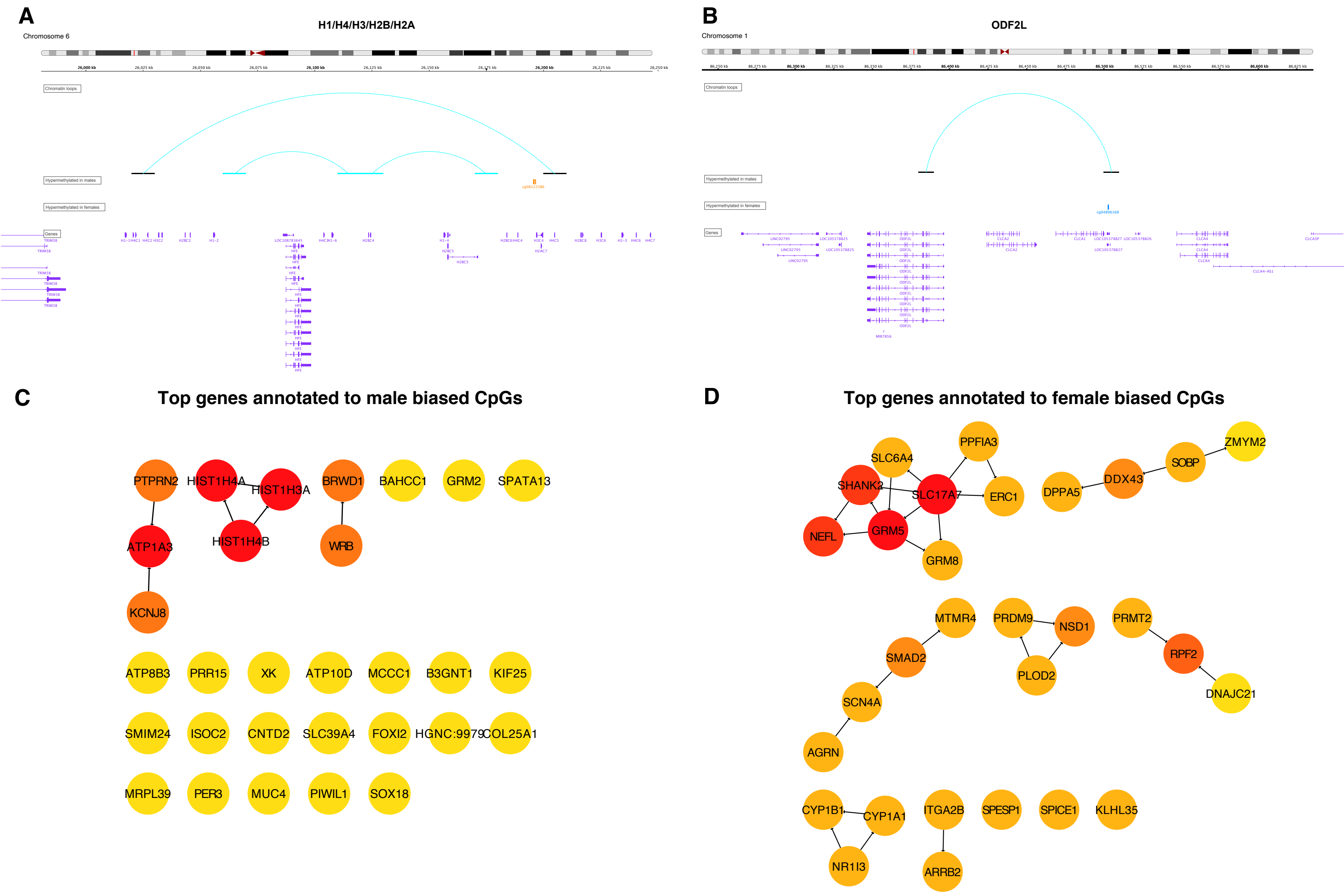
A
H1/H4/H3/H2B/H2A
Chromosome 6
Chromatin loops
Hypermethylated in males
Hypermethylated in females
Genes
B
ODF2L
Chromosome 1
Chromatin loops
Hypermethylated in males
Hypermethylated in females
Genes
C
Top genes annotated to male biased CpGs
PTPRN2
HIST1H4A
HIST1H3A
BRWD1
BAHCC1
GRM2
SPATA13
ATP1A3
HIST1H4B
WRB
KCNJ8
ATP8B3
PRR15
XK
ATP10D
MCCC1
B3GNT1
KIF25
SMIM24
ISOC2
CNTD2
SLC39A4
FOXI2
HGNC:9979
COL25A1
MRPL39
PER3
MUC4
PIWIL1
SOX18
D
Top genes annotated to female biased CpGs
PPFIA3
ZMYM2
SLC6A4
SOBP
SHANK2
SLC17A7
ERC1
DDX43
DPPA5
NEFL
GRM5
GRM8
MTMR4
PRDM9
NSD1
PRMT2
SMAD2
RPF2
PLOD2
SCN4A
DNAJC21
AGRN
CYP1B1
CYP1A1
ITGA2B
SPESP1
SPICE1
KLHL35
NR1I3
ARRB2
